# Supplementary material for: Inter-monitor reliability and validity of the fibion accelerometers in a laboratory-based study of functional activities
Source: PLoS One. 2025 May 20;20(5):e0324555. doi: 10.1371/journal.pone.0324555 (PMC12091727; doi:10.1371/journal.pone.0324555)
Supplement: S1 File — Supplementary Table 1: Reliability of activity type classification accuracy by the Fibion accelerometer for all activities combined for different accelerometer locations and same-side comparisons. Supplementary Table 2: Reliability of activity intensity classification by the Fibion accelerometer for all activities combined included in the study for different accelerometer locations and same-side comparisons. (PDF) [file pone.0324555.s001.pdf]

Supplementary Table 1. Reliability of activity type classification accuracy by the Fibion accelerometer for all activities combined for different accelerometer locations and same side comparisons.

	Right vs. left			Right side only		
	Pocket	Proximal thigh	Distal thigh	Pocket vs. proximal thigh	Pocket vs. distal thigh	Proximal vs. distal thigh
**Kappa value**	0.79*	0.82*	0.82*	0.76*	0.70*	0.81*
**% Agreement**	85.2	87.3	88.8	83.3	79.9	86.7

*p-value <0.001

Supplementary Table 2. Reliability of activity intensity classification by the Fibion accelerometer for all activities combined included in the study for different accelerometer locations and same-side comparisons.

	Right vs. Left			Right side only		
	Pocket	Proximal thigh	Distal thigh	Pocket vs. proximal thigh	Pocket vs. distal thigh	Proximal vs. distal thigh
**Weighted Kappa value**	0.71*	0.76*	0.71*	0.66*	0.63*	0.68*
**% agreement**	91.2	92.5	91.9	90.0	89.3	91.1

*p-value < 0.001
